# Protective effect of regular physical activity on major depressive episodes in patients with early stages of chronic kidney disease

**DOI:** 10.1080/0886022X.2017.1361833

**Published:** 2017-08-14

**Authors:** Fu-Xiang Zhu, Xiang-Yang Zhang, Xiao-Kai Ding, Bin Han

**Affiliations:** a Department of Nephrology, First Affiliated Hospital of Jiaxing University, Jiaxing, China;; b Menninger Department of Psychiatry and Behavioral Sciences, Baylor College of Medicine, Houston, TX, USA;; c Department of Nephrology, First Affiliated Hospital of Wenzhou Medical University, Wenzhou, China

**Keywords:** Depression, chronic kidney disease, physical activity, China, inflammation

## Abstract

Previous studies have demonstrated an association between physical activity (PA) and depression in diverse population. The purpose of our study is to examine if PA within the recommended level over time is associated with major depressive episode (MDE) in patients with early stages of chronic kidney disease (CKD) in Mainland China. Patients with stages 2–5 CKD not receiving dialysis were enrolled from a nephrology outpatient clinic between May 2014 and February 2016. Based on the patterns of PA over time, all patients were divided into four groups: persistently active, from inactive to active, from active to inactive, and persistently inactive. An MDE was diagnosed by using the Diagnostic and Statistical Manual of Mental Disorders (Fourth Edition)-based the Mini International Neuropsychiatric Interview. Among 150 patients, 34 had a current MDE (22.7%) and 116 did not (77.3%). After multivariable adjustment, patients being persistently active had significantly lower odds of having an MDE (odds ratio 0.102, 95% confidence interval, 0.022–0.467, *p* = .003) compared with those who were persistently inactive. Additionally, patients with diabetes mellitus had significantly higher odds of having an MDE (odds ratio 4.287, 95% confidence interval, 1.473–12.483, *p* = .008) compared with those without diabetes mellitus. Our results suggest a protective effect of regular PA on MDE in patients with early stages of CKD in Mainland China.

## Introduction

Depression is prevalent in patients with chronic kidney disease (CKD), affecting up to 21% of patients even before receiving maintenance dialysis [1]. The presence of depression is associated with increased risk of progression to dialysis, reduced quality of life, hospitalization, and death [2–4]. The number of patients with early stages of CKD is much higher than that of patients receiving dialysis and has been increasing steadily for decades [5,6]. Therefore, early recognition and diagnosis of depressive complication is of great importance in better outcomes.

Physical activity (PA) helps improve physical performance and mental function. A growing number of studies have suggested that PA was associated with a lower risk of depression in diverse population [7–10]. Moreover, studies of physical exercise intervention in depressive disorders demonstrated positive effects of PA on depression [11,12]. In CKD patients PA is strikingly below recommended levels, which is related to their poor outcomes [13,14]. Recent two studies have demonstrated a significant association between current exercises and depressive symptoms in patients with pre-dialysis CKD [15,16]. But the cross-sectional nature of these studies limited the ability to confirm causality between current exercises and depressive symptoms. Additionally, diagnosis of depression in these two studies was ascertained just using self-report measures, not Structured Clinical Interview for Diagnostic and Statistical Manual of Mental Disorders (Fourth Edition). Up to now, no study has explored the association between PA and depressive complication of CKD in Mainland China. A cohort of patients with CKD not yet started on dialysis was studied to determine if there was an association between PA within the recommended level over time and major depressive episode (MDE), based on Diagnostic and Statistical Manual of Mental Disorders (Fourth Edition).

## Methods

### Participants

Patients who are in Mainland China were recruited from the First Affiliated Hospital of Jiaxing University, Zhejiang Province, Nephrology Outpatient Clinic between May 2014 and February 2016. The study was in accordance with the declaration of Helsinki. The Ethics Committee of the First Affiliated Hospital of Jiaxing University approved the study and informed written consent was obtained from all participating patients. Patients with CKD (stages 2–5), defined as an estimated glomerular filtration rate (eGFR) of less than 90 mL/min/1.73 m^2^ for three months, using the four-variable modification of diet in renal disease formula [17], were included. Patients with stage 2 CKD (eGFR of 60–89 mL/min/1.73 m^2^) had to have other evidence of kidney damage manifest by either pathological abnormality of kidney on biopsy or markers of kidney damage present for at least three months [18]. Exclusion criteria were initiation of dialysis or kidney transplantation, and no health care power of attorney to sign consent.

### Assessment of depression

All participants underwent a structured clinical interview to ascertain the presence of an MDE according to the Mini International Neuropsychiatric Interview (MINI), a widely used interview based on the Diagnostic and Statistical Manual of Mental Disorders (Fourth Edition) [19] which was used as the gold standard for the diagnoses of major depressive disorder (MDD), as well as other psychiatric diagnosis. The MINI, which takes 30–45 min to complete, was performed by 1 of 2 trained researchers blinded to patients’ medical records.

### Physical activity level

According to the international guidelines recommendations that adults participate in daily PA [20], two questions about PA were as follows: (1) whether you regularly participated in PA, sports or exercise, lasting ≥150 min/week before being diagnosed with CKD; (2) whether you regularly participate in PA, sports or exercise, lasting ≥150 min/week now. ‘Yes’ was defined as an ‘active’, otherwise as ‘inactive’. The summary indices used to calculate the cutoff value for meeting the recommended PA level are based on the work of Kurtze et al. [21] on the validity of self-reported PA. Those who were active at both phases were ‘stable active’, those who decreased from active and became inactive were classified as ‘decreasing’. Those who moved from being inactive to active were defined as ‘increasing’, and those who were inactive at both phases were ‘stable inactive’.

### Data collection

Demographic and clinical data (e.g., age, sex, smoking status, alcohol use, education, employment status, religion, habitation, body weight and height, hemoglobin, albumin, creatinine and eGFR) were collected from the centralized patient record. A comorbidity index, comprising a sum of comorbid conditions of interest, was calculated for each participant, as in previous study by Evans et al. [22] Comorbid medical illness included hypertension, diabetes mellitus, congestive heart failure, coronary artery disease, cerebrovascular disease, peripheral vascular disease, lung disease, liver disease, non-skin malignancy, and infection with human immunodeficiency virus (HIV). A current or past history of any psychiatric disorders including major depressive disorder was also recorded at enrollment. Any current or past alcohol abuse collected from the centralized patient record system was coded as alcohol abuse.

### Statistical analysis

The clinical variables of all patients were compared using the Pearson *χ* ^2^ test, one-way ANOVA, Mann–Whitney *U-*test and Fisher’s exact test, as appropriate. The *post hoc* Tukey test was applied to assess differences in two-group comparisons, when ANOVA showed significant differences between the groups. Bonferroni corrections were used to each test to adjust for multiple testing. Logistic regression model including all factors significantly different in the univariate analysis was performed to examine the association between PA over time and a current MDE in patients with early stage of CKD. The result was expressed as adjusted odds ratios (OR) with the corresponding 95% confidence intervals (CI). To evaluate if sex and age modified the association, we computed two interaction terms (PA pattern*sex, and PA pattern*age), which were analyzed separately in a fully adjusted model. Data were analyzed using SPSS for Windows, version 17.0 (SPSS Inc., Chicago, IL, USA). Statistical significance was defined as *p* < .05.

## Results

### Baseline characteristics of study samples

Of 197 patients who were approached for the current study, 155 (78.7%) agreed to sign consent and 42 (21.3%) refused to participate. Five patients did not complete the interview and were excluded. The final analysis included 150 participants (age 62.3 ± 7.3 years; 40% female). The baseline data of participants and nonparticipants, such as sex, age, BMI, CKD stage, presence of diabetes, and so on were similar. Among participants, more than a third had diabetes. The disease duration of CKD was 3.24 ± 1.45 years. These prevalences were 12.7% for CKD stage 2, 36.0% for stage 3, 32.0% for stage 4, and 19.3% for pre-dialysis stage 5.

### Comparison of the cohort based on a current major depressive episode

Baseline descriptive data are shown in Table 1. Between the categories of PA pattern, there were significant differences in single household (*p* = .006), unemployed (*p* < .001), and diabetes mellitus (*p* < .001). Of 150 participants, 34 (22.7%) had a current MDE and 116 (77.3%) did not. None of them received antidepressant treatment during study procedures. The prevalence of depression was 12.2% (*n* = 6) among those who were persistently active, 24.0% (*n* = 6) among those who changed from being inactive to being active, 27.9% (*n* = 12) in those who changed from being active to inactive, and 30.3% (*n* = 10) among those who maintained being persistently inactive, but Pearson *χ* ^2^ test indicated that these differences were not statistically significant (*p* = .184).

**Table 1.** Unadjusted baseline characteristics by patterns of PA habits over time.

	Stable active (*n* = 49)	From inactive to active (*n* = 25)	From active to inactive (*n* = 43)	Stable inactive (*n* = 33)	*p* value
Age, mean (SD), y	61.0 (6.8)	62.4 (4.9)	63.5 (8.2)	62.7 (7.3)	.434
Female	17 (34.7)	9 (36.0)	17 (39.5)	17 (51.5)	.463
BMI, mean (SD), kg/m^2^	21.4 (2.4)	22.0 (2.3)	21.5 (2.4)	22.0 (2.7)	.579
Education, mean (SD), y	8.8 (2.5)	8.6 (1.9)	9.4 (3.3)	8.9 (2.6)	.860
Religion (yes)	38 (77.6)	22 (88.0)	35 (81.4)	27 (81.8)	.753
Single household	18 (36.7)	3 (12.0)	10 (23.3)	2 (6.1)	.006
Unemployed	17 (34.7)	21 (84.0)	23 (53.5)	30 (90.9)	<.001
Current smoking	14 (28.6)	11 (44.0)	20 (46.5)	15 (45.5)	.261
Alcohol abuse	6 (12.2)	2 (8.0)	4 (9.3)	7 (21.2)	.374
Diabetes mellitus	6 (12.2)	3 (12.0)	22 (51.2)	26 (78.8)	<.001
Previous psychiatric history	4 (8.0)	2 (8.0)	3 (7.0)	5 (15.2)	.622
Comorbidity					.890
≤1	7 (20.4)	4 (16.0)	9 (20.9)	5 (15.2)	
≥2	39 (79.6)	21 (84.0)	34 (79.1)	28 (84.8)	
Duration of CKD, mean (SD), y	2.99 (1.33)	3.40 (1.53)	3.30 (1.50)	3.40 (1.52)	.637
CKD by stage					.623
2	8 (16.3)	5 (20.0)	4 (9.3)	2 (6.1)	
3	18 (36.7)	10 (40.0)	16 (37.2)	10 (30.3)	
4	16 (32.7)	7 (28.0)	14 (32.6)	11 (33.3)	
5	7 (14.3)	3 (12.0)	9 (20.9)	10 (30.3)	
SBP, mean (SD), mmHg	135 (13.2)	135 (14.5)	141 (18.2)	138 (16.4)	.426
Hemoglobin, mean (SD), g/dL	10.3 (2.1)	10.0 (2.3)	9.9 (2.0)	9.6 (2.1)	.540
Albumin, mean (SD), g/dL	3.0 (0.3)	3.0 (0.3)	3.0 (0.3)	2.9 (0.4)	.184
Creatinine, mean (SD), mg/dL	4.5 (0.9)	4.6 (1.0)	4.5 (0.9)	4.7 (1.2)	.830
Estimated GFR, mean (SD), mL/min	38.0 (9.8)	33.8 (10.7)	37.0 (9.7)	37.8 (8.4)	.251

Data are presented as mean (SD) and numbers (%) if not otherwise stated.

PA: physical activity; SD: standard deviation; BMI: body mass index; CKD: chronic kidney disease; SBP: systolic blood pressure; GFR: glomerular filtration rate.

### Variables associated with a current major depressive episode

In the logistic analysis, with the category ‘persistently inactive’ taken as the reference group and adjustment for single household, unemployed, and diabetes mellitus, those being persistently active showed a reduced likelihood (OR 0.102, 95% CI, 0.022–0.467, *p* = .003) of having a current MDE compared with those who were persistently inactive. Moreover, participants with diabetes mellitus had significantly increased odds of having MDE (OR 4.287, 95% CI, 1.473–12.483, *p* = .008) compared with those without diabetes mellitus (Table 2). There was no significant interaction between age and pattern of PA (*p* = .459) and between sex and pattern of PA (*p* = .732).

**Table 2.** Odds ratios (or) and 95% confidence intervals (CI) of MDE by changes in PA patterns over time.

Variables	OR	95%CI	*p* value
Patterns of PA habits			
Stable active	0.102	(0.022–0.467)	.003
From inactive to active	0.266	(0.065–1.098)	.065
From active to inactive	0.511	(0.155–1.683)	.270
Single household	0.600	(0.206–1.752)	.350
Unemployed	1.016	(0.391–2.643)	.974
Diabetes mellitus	4.287	(1.473–12.483)	.008

PA: physical activity; MDE: major depressive episode.

## Discussion

To our knowledge, this is by far the first study exploring the possible association between PA over time and a current MDE in patients with pre-dialysis CKD in Mainland China. Our results suggest a protective effect of stable PA on depressive complication of patients with early stages of CKD.

In the present study, we found that 22.7% of patients with early stages of CKD had a current MDE, which is consistent with previous studies [1,15]. Our results also demonstrated that diabetes mellitus were significantly associated with an MDE. Numerous studies have indicated a higher prevalence of depression in non-CKD populations with diabetes compared with the general population [23–25]. Moreover, several prospective cohort studies showed that patients with diabetes mellitus had a greater risk of developing increased depressive symptoms [26,27]. No significant association between the depressive complication and other variables was found, including unemployed, psychiatric history, and hemoglobin level, although previous studies have provided controversial results on these aspects.

Importantly, our study demonstrated a protective effect of stable PA on a current MDE among patients with early stages of CKD in Mainland China. The exact mechanisms responsible for the depressive complication are unknown. It has been suggested that participation in moderate exercise may improve immune function and exert anti-inflammatory effects [28–30]. CKD is associated with profound changes in innate and adaptive immunity, which predispose CKD patients to infectious complications and chronic inflammation. Viana et al. [31] reported that moderate intensity exercise exert ant-inflammatory effects in patients with pre-dialysis CKD. Accumulating evidence suggests inflammatory response may play an important role in the pathogenesis of depression [32–34]. Therefore, one of the mechanisms linking PA to depression among patients with early stages of CKD is the anti-inflammatory effects of PA. Additionally, those who were persistently active are able to enhance their social network, thereby having more resources with which to face psychological stress associated with CKD management.

There are several limitations in the present study. First, the rigid inclusion criteria resulted in a small sample size, which reduced the statistical power of the study. Second, due to difficulty in collecting the details on the type of PA, we could not analyze the possible association between the type of PA and depression in patients with early stages of CKD. Third, it was a retrospective investigation, therefore, observer bias and recall error should not be excluded. Lastly, patients in our sample came from only one clinic. Therefore, our findings may not be readily generalized to other CKD patients.

## Conclusions

In summary, in spite of these limitations, we observed an important protective effect of regular PA on an MDE in patients with early stage of CKD in Mainland China. Further randomized controlled trials should be encouraged to examine whether physical exercises intervention holds promise the treatment of depression in patients with early stage of CKD.
